# Short-term outcomes after open versus robot-assisted repair of ventral hernias: a nationwide database study

**DOI:** 10.1007/s10029-023-02923-8

**Published:** 2023-11-30

**Authors:** N. A. Henriksen, F. Helgstrand, K. K. Jensen

**Affiliations:** 1 Department of Gastrointestinal and Hepatic Diseases, Herlev Hospital, University of Copenhagen, Borgmester Ib Juuls Vej 1, 2730 Herlev, Denmark; 2 Department of Surgery, Zealand University Hospital, Koege, Denmark; 3 Department of Surgery and Transplantation, Rigshospitalet, Copenhagen, Denmark

**Keywords:** Surgical-site occurrence, Length of stay, Readmission, Reoperation, Incisional hernia, Umbilical hernia

## Abstract

**Purpose:**

The robotic platform is widely implemented; however, evidence evaluating outcomes of robotic ventral hernia repair is still lacking. The aim of the study was to evaluate the short-term outcomes after open and robot-assisted repair of primary ventral and incisional hernias.

**Methods:**

Nationwide register-based cohort study with data from the Danish Ventral Hernia Database and the National Danish Patients Registry was from January 1, 2017 to August 22, 2022. Robot-assisted ventral hernia repairs were propensity score matched 1:3 with open repairs according to the confounding variables defect size, Charlson comorbidity index score, and age. Logistic regression analyses were performed for factors associated with length of stay > 2 days, readmission, and reoperation within 90 days.

**Results:**

A total of 528 and 1521 patients underwent robot-assisted and open repair, respectively. The mean length of hospital stay in days was 0.5 versus 2.1 for robot-assisted and open approach, respectively (*P* < 0.001) and open approach was correlated with risk of length of stay > 2 days (OR 23.25, CI 13.80–39.17, *P* < 0.001). The incidence of readmission within 90 days of discharge was significantly lower after robot-assisted repair compared to open approach (6.2% vs. 12.1%, *P* < 0.001). Open approach was independently associated with increased risk of readmission (OR 21.43, CI 13.28–39.17, *P* = 0.005, *P* < 0.001).

**Conclusion:**

Robot-assisted ventral hernia repair is safe and feasible and associated with shorter length of stay and decreased risk of readmission compared with open ventral hernia repair.

## Introduction

The choice between an open or a laparoscopic approach for ventral hernia repair has been discussed for decades. Though it seems clear that the two approaches are comparable considering recurrence rates, there are significantly fewer wound complications and faster recovery after a laparoscopic approach [1–6]. Recent large registry studies have confirmed these findings and concluded that readmission and reoperation rates were also decreased after laparoscopic repair of medium sized ventral hernias [7–9].

Traditionally, a laparoscopic ventral hernia repair has been performed with an intraperitoneal onlay mesh (IPOM). With this procedure, a coated mesh is placed intra-peritoneally and fixated with tackers [10]. The procedure is easy to learn with good short-term outcomes; however, concerns about long-term complications to the intraperitoneal mesh and chronic pain due to mesh fixations have arisen [11, 12]. Therefore, alternative laparoscopic approaches avoiding intraperitoneal mesh and tack fixation have evolved the last decade [13–16].

With the introduction of robot-assisted laparoscopy, minimally invasive ventral hernia repair has become easier due to articulation of the robotic arms, making dissecting, and suturing upwards at the abdominal wall simpler. Thus, patients that previously mainly were offered open surgery now can have the benefit of minimal invasive surgery. Furthermore, robot-assisted laparoscopy also makes pre-peritoneal or retro-muscular mesh placement possible compared to traditional laparoscopic ventral hernia repair, where intraperitoneal onlay mesh placement for many years have been standard [17].

Some studies have compared open and robot-assisted ventral hernia repair and concluded that length of stay and recovery are shorter for patients receiving the robot-assisted ventral hernia repair [18–20]. Randomized controlled trials and larger registry studies are still needed to evaluate outcomes after open and robot-assisted ventral hernia repair on prospective and large-scale basis.

It is our hypothesis that robot-assisted ventral hernia repair is feasible with low complication rate and improved postoperative outcomes compared with open ventral hernia repair. Therefore, this nationwide database study was undertaken with the aim of evaluating short-term outcomes after open and robot-assisted repair of primary ventral and incisional hernia. The primary outcome was length of stay > 2 days, whereas secondary outcomes included readmission and operative re-intervention within 90 days postoperatively.

## Methods

This was a nationwide register-based cohort study of patients undergoing open and robot-assisted repair of a primary ventral or an incisional hernia. Patients were identified using the Danish Ventral Hernia database, which is a national database including all patients undergoing ventral hernia repair in Denmark [21]. All surgeons are obliged to report patient data and details of the surgical procedure, and follow-up is achieved using the nationwide National Patient Register, which holds details of all hospital-related contacts including readmission and operative re-intervention [22]. The study period was January 1, 2017, to August 22, 2022 and was chosen due to the introduction of robot-assisted surgery in this period. The exposure of interest was surgical approach, defined as open or robot-assisted ventral hernia repair.

To reduce heterogeneity, only open repairs with a mesh placement in the pre-peritoneal or retro-muscular plane were included as these planes were also used in robot-assisted repairs. To reduce the risk of bias, patients undergoing robot-assisted ventral hernia repair were propensity score matched in a 1:3 ratio with patients undergoing open ventral hernia repair and were matched according to the confounding variables horizontal fascial defect size, Charlson comorbidity index score, and age. Propensity score matching reduces the risk of selection bias in observational studies and is a statistical way of adjusting for the non-randomized setting [23]. The study was written in accordance with the STROBE guidelines for the reporting of observational cohort studies [24].

### Variables

The primary outcome was postoperative length of hospital stay (LOS), which was given both as a numerical variable and a binary outcome defined as LOS > 2 (yes/no). Further outcomes reported were readmission to the hospital and operative re-intervention within 90 days. Demographic variables included were gender, age, smoking status (current smoker/non-smoker), body mass index (BMI), and Charlson comorbidity index score. The intraoperative variables included were horizontal and vertical defect sizes at the time of surgery, plane for mesh placement, and type of mesh fixation.

### Statistics

The propensity score matching was performed in a 1:3 ratio, using the nearest-neighbor method and 0.10 caliper. Numerical variables were given as mean (IQR) and compared across groups using Student’s *t*-test or Mann–Whitney according to data distribution, whereas categorical variables were reported as *n* (%) and compared using chi-squared test.

Primary outcome, LOS > 2 days, was further examined using multivariable logistic regression analysis, including the exposure of interest (open or robotic approach) and the a priori selected confounders gender, age, comorbidity, horizontal fascial defect size, and type of hernia (primary or incisional). The results of the multivariable analysis were presented as odds ratio (OR) and 95% confidence interval (CI) along with *P *value. The secondary outcomes (hospital readmission within 90 days postoperative and operative re-intervention within 90 days postoperative) were also examined using an identical approach.

*P *values < 0.05 was considered statistically significant. All analyses in the current study were performed using R version 4.2.2 (R Foundation for Statistical Computing, Vienna, Austria).

### Ethics

Patient consent is not necessary as reporting to clinical databases is mandatory in Denmark. Permission to conduct the study was given by the board of the Danish Hernia Database and the Regional Data Protection Agency of the Capital Region, Denmark (REG-138-2018).

## Results

**Fig. 1:**
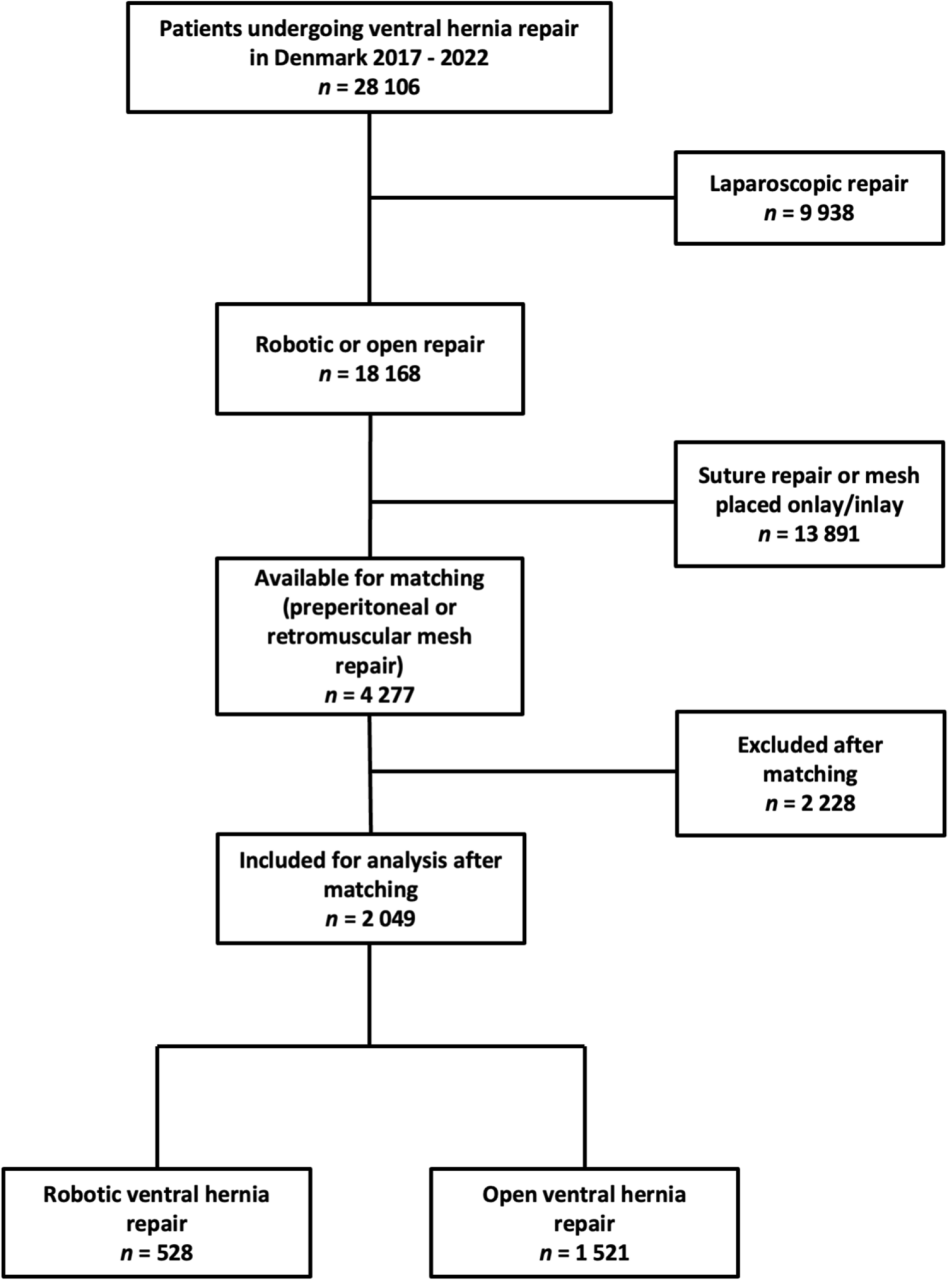
Flowchart of included patients

During the study period, a total of 28,106 patients underwent ventral hernia repair. Of these, 559 underwent robot-assisted and 17,609 open repairs. After propensity score matching, a total of 2049 patients were included in the study, of whom 528 and 1521 patients underwent robot-assisted and open repair, respectively (Fig. 1). The yearly incidence of robot-assisted repairs increased during the 6-year inclusion period. Patients in the two groups were comparable in terms of age and gender distribution, comorbidities, and hernia defect size (Table 1). There were significantly more smokers and obese patients receiving a robot-assisted repair, whereas more patients were operated for an incisional hernia in the group of patients receiving an open repair (Table 1).

**Table 1:** Characteristics of patients undergoing robot-assisted or open ventral hernia repair in Denmark from 2017 to 2022

Variable	Robot-assisted ventral hernia repair (*n* = 528)	Open ventral hernia repair (*n* = 1521)	*P* value
Age			
< 45	90 (17.0)	204 (13.4)	0.152
45–60	193 (36.6)	555 (36.5)	
> 60–75	202 (38.3)	611 (40.2)	
> 75	43 (8.1)	151 (9.9)	
Gender			
Male	302 (57.2)	945 (62.1)	0.051
Female	226 (42.8)	576 (37.9)	
Tobacco smoking	88 (16.7)	176 (11.6)	0.003
Charlson comorbidity index score			
0	300 (56.8)	775 (51.0)	
1	89 (16.9)	286 (18.8)	
> 1	139 (26.3)	460 (30.2)	0.066
Body mass index (kg/m^2^)			
Median (range)	30.3 (20.6–50.8)	29.1 (17.1–58.1)	< 0.001
Year of surgery			
2017	29 (5.5)	278 (18.3)	< 0.001
2018	81 (15.3)	300 (19.7)	
2019	99 (18.8)	246 (16.2)	
2020	97 (18.4)	287 (18.9)	
2021	144 (27.3)	290 (19.1)	
2022	78 (14.8)	120 (7.9)	
Type of hernia			
Primary ventral	351 (66.5)	930 (61.1)	0.033
Incisional	177 (33.5)	591 (38.9)	
Hernia defect size, cm			
Mean (sd)			
Horizontal	3.9 (4.6)	4.1 (5.3)	0.462
Vertical	4.4 (5.1)	5.1 (6.8)	0.039
Mesh placement			
Preperitoneal	109 (20.6)	755 (49.6)	< 0.001
Retromuscular	419 (79.4)	766 (50.4)	
Mesh fixation			
Self-fixating mesh	377 (71.4)	259 (17.0)	< 0.001
Suture	33 (6.2)	996 (65.5)	
Tacks	5 (0.9)	12 (0.8)	
Glue	22 (4.2)	6 (0.4)	
None	76 (14.4)	207 (13.6)	
Other	4 (0.8)	24 (1.6)	
*Postoperative outcomes*			
Length of stay			
Median (range)	0.5 (0, 32)	2.1 (0, 29)	< 0.001
Length of stay > 2 days	29 (5.7)	550 (37.3)	< 0.001
Readmission within 90 days	33 (6.2)	184 (12.1)	0.001
Reoperation within 90 days	9 (1.7)	52 (3.4)	0.065

The mesh was placed in the retro-muscular position more frequently in the robot-assisted repairs (79.4%, 419/528) compared with the open repairs (50.4%, 766/1521), *P* < 0.001. A self-fixating mesh was the most frequently used mesh type for robot-assisted repairs (71.4%, 377/528), whereas the mesh was fixated with sutures in most of the open procedures (65.5%, 996/1521) (Table 1).

The mean length of hospital stay was significantly shorter after robot-assisted repair compared to open repair (mean days 0.5 vs. 2.1, *P* < 0.001), and the rate of length of stay > 2 days was similarly significantly shorter after robot-assisted repair (5.7% vs. 37.3%, *P* < 0.001). After multivariable analysis, open approach was significantly associated with an increased risk of length of stay > 2 days (OR 21.43, CI 13.80–39.17, *P* < 0.001). Other factors associated with increased risk of prolonged length of stay were female gender, comorbidity, incisional compared to primary ventral hernia repair, and increasing horizontal defect size (Table 2).

**Table 2:** Multivariable logistic regression analysis of factors associated with increased risk of postoperative length of hospital stay > 2 days after robot-assisted and open ventral hernia repair

	OR	CI	*P* value
Age			
< 45	1.00		
45–60	1.21	0.76–1.95	0.423
> 60–75	0.93	0.57–1.52	0.770
> 75	1.64	0.91–2.97	0.100
Female gender	1.48	1.12–1.96	0.006
Charlson score			
0	1.00		
1	1.32	0.90–1.93	0.152
> 1	1.56	1.12–2.18	0.009
Horizontal defect			
< 4 cm	1.00		
4–8 cm	12.97	9.32–18.05	< 0.001
> 8 cm	23.25	13.28–39.17	< 0.001
Surgical approach			
Robot-assisted	1.00		
Open	21.43	13.28–34.60	< 0.001
Hernia type			
Primary	1.00		
Incisional	4.97	3.73–6.64	< 0.001

The incidence of readmission within 90 days of discharge was significantly lower after robot-assisted repair compared to open approach (6.2% vs. 12.1%, *P* < 0.001) (Table 1). Superficial wound complication including hematoma was the most frequent cause of readmission and was significantly higher after open repair 4.1% (63/1521) compared to robot-assisted approach 0.7% (4/528),* P* < 0.001 (Table 3). After multivariable analysis, open approach was associated with a significantly increased risk of readmission (OR 1.95, CI 1.32–2.88, *P* = 0.001). Other factors significantly associated with increased risk of readmission were comorbidity and increasing horizontal defect size above 8 cm (Table 4).

**Table 3:** Causes for readmission within 90 days after robot-assisted and open ventral hernia repair

	Robot-assisted (*n* = 33)	Open (*n* = 184)
Pain	12	34
Superficial wound complication	4	48
Hematoma	0	15
Bowel obstruction	2	1
Constipation	0	5
Hepatobiliary disease	0	3
Other gastrointestinal disease	0	12
Urogenital disease	3	6
Pulmonary disease	3	7
Cardiac disease	2	6
Neurological disease	0	2
Pain in extremities and back	1	4
Endocrine disease	0	4
Psychiatric disease	0	5
Other	6	32

**Table 4:** Multivariable logistic regression analysis of factors associated with increased risk of readmission within 90 days after robot-assisted and open ventral hernia repair

	OR	CI	*P* value
Age			
< 45	1.00		
45–60	1.18	0.72–1.91	0.506
> 60–75	0.87	0.52–1.44	0.579
> 75	1.06	0.56–2.00	0.859
Female gender	1.22	0.91–1.65	0.178
Charlson score			
0	1.00		
1	1.48	1.02–2.16	0.040
> 1	1.30	0.91–1.86	0.149
Horizontal defect			
< 4 cm	1.00		
4–8 cm	1.53	1.06–2.20	0.023
> 8 cm	2.28	1.42–3.64	0.001
Surgical approach			
Robot-assisted	1.00		
Open	1.95	1.732–2.88	0.001
Hernia type			
Primary	1.00		
Incisional	1.09	0.78–1.52	0.611

The incidence of surgical re-intervention within 90 days of ventral hernia repair was insignificantly lower after robot-assisted repair compared to open approach (1.7% vs. 3.4%, *P* = 0.06) (Table 1). The most frequent cause of surgical re-intervention was operation for surgical-site infection with comparable rates in open (0.9%, 14/1521) and robot-assisted repairs (0.4%, 2/528),* P* = 0.223 (Table 5). After multivariable analysis, open approach was not significantly associated with increased risk of surgical re-intervention (OR 2.02, CI 0.98–4.14, *P* = 0.05). No other variables were significantly associated with increased risk of surgical re-intervention (Table 6).

**Table 5:** Causes for surgical re-intervention within 90 days after robot-assisted and open ventral hernia repair

	Robot-assisted (*n* = 9)	Open (*n* = 52)
Reoperation for superficial wound infection	2	18
Reoperation for deep infection	0	0
Reoperation for early recurrence	3	4
Reoperation for bowel obstruction	0	2
Bowel resection	0	4
Laparoscopy	0	2
Laparotomy	0	6
Other gastrointestinal surgery	3	8
Endoscopy	1	8

**Table 6:** Multivariable logistic regression analysis of factors associated with increased risk of surgical re-intervention within 90 days after robot-assisted and open ventral hernia repair

	OR	CI	*P* value
Age			
< 45	1.00		
45–60	1.06	0.46–2.46	0.891
> 60–75	0.80	0.32–1.95	0.618
> 75	1.05	0.36–2.10	0.925
Female gender	1.36	0.80–2.30	0.258
Charlson score			
0	1.00		
1	1.28	0.63–2.59	0.491
> 1	1.55	0.83–2.90	0.171
Horizontal defect			
< 4 cm	1.00		
4–8 cm	0.98	0.49–1.95	0.958
> 8 cm	1.17	0.46–2.98	0.743
Surgical approach			
Robot-assisted	1.00		
Open	2.02	0.98–4.14	0.055
Hernia type			
Primary	1.00		
Incisional	1.02	0.56–1.85	0.951

## Discussion

This is the first nationwide database study evaluating short-term outcomes after robot-assisted and open ventral hernia repair. Length of stay was significantly shorter for robot-assisted ventral hernia repairs, and the risk of readmission within 90 days was significantly decreased compared to open repair.

This study included patients from the first era of robot-assisted ventral hernia repairs in Denmark. Despite an expected learning curve for a new procedure, robot-assisted ventral hernia repair was still associated with significantly better outcomes than open repair, suggesting that robot-assisted ventral hernia repair is safe and feasible with overall good outcomes. Interestingly, the numbers of robot-assisted repairs increased over the 6-year period with the highest number of procedures registered in 2021, reflecting that increasingly more hospitals are getting access to robotic platforms in Denmark.

The postoperative hospital stay after robot-assisted repair was significantly shorter after robot-assisted repair, but the specific reasons for this were not assessed in the current study. One probable explanation is the reduced pain after a minimally invasive procedure with no painful mesh fixation [25]. Further, patients undergoing open repair of medium or large ventral hernia most often require either epidural catheter or a peripheral nerve block leading to prolonged hospitalization [26]. The reason we chose to define prolonged hospitalization as LOS > 2 days was that day-case surgery in Denmark is not performed in patients who are living alone or those with severe cardiopulmonary comorbidity. Thus, 1 or 2 days in the hospital postoperatively may be explained by these factors, whereas more than 2 days LOS was considered related to the type of approach. Although enhanced recovery protocols are being implemented for open repair of large ventral hernia, there are no reports of such protocols after laparoscopic or robot-assisted ventral hernia repair currently [27].

The reduced incidence of readmission after robot-assisted compared to open ventral hernia repair was primarily due to a reduction in the surgical-site occurrences. Interestingly, more than one-third of the patients requiring readmission after robotic repair were due to pain, as opposed to less than one-fifth after open repair. This may be a direct consequence of the significantly less postoperative length of stay after robotic repair as patients undergoing open repair had more time in the hospital postoperatively for optimal analgetic treatment. Naturally, a reduction in complications is desirable; however, for patients undergoing hernia repair, this reduction of surgical-site complications is crucial due to the strong association with hernia recurrence [28]. Especially, patients with incisional hernias are known to have an increased risk of postoperative complications, and the combination of minimally invasive approach and retro-muscular mesh placement thus may be the optimal way to reduce the long-term risk of hernia recurrence.

There was a tendency toward a decreased rate of surgical re-interventions within 90 days in the uni- and multivariable analyses though statistical significance was not reached. Fortunately, the overall rate of surgical re-intervention was low and hypothetically with a larger a sample size. This would have become significant.

Using the robot for ventral hernia repairs seems beneficial in improving early outcomes, both when compared to the traditional laparoscopic IPOM technique and open repairs [20, 29]. But one proposed downside to robot-assisted hernia repair is the increased costs associated with this approach. Besides the one-time cost of obtaining a robotic platform, maintenance and instruments with limited procedural capacity come at a higher expense compared to open repair [18, 30]. However, the results of the current study may nuance this debate, as longer length of stay and readmissions to the hospital are associated with significant increases in procedural expenses [31, 32]. As no data on hospital costs are available from the current study, future studies of the costs associated with ventral hernia repair thus should take the postoperative complications and readmissions into account.

This study is strengthened by the fact that it is including nationwide prospectively registered procedures of real-world data. However, there are limitations to the study. Given the fact, that the study is a database study and not randomized, there may be a bias in patient selection to the two procedures. However, there were more smoking and obese patients receiving robot-assisted repairs, which are factors normally associated with increased risk of complications and yet outcomes in the robotic group were better. Another potential bias could be that surgeons performing robot-assisted hernia repairs may hypothetically be more well-trained and experienced hernia surgeons, which could hypothetically improve patient outcomes. Furthermore, the study included both primary ventral and incisional hernia repairs, and as these two patient groups are very different by nature, one could argue, that these should not be included in the same study. It was decided to do so, to get enough power to perform the study now. Long-term outcomes such as recurrence were not included, as robot-assisted ventral hernia repair is still a relatively new procedure in Denmark, and it is too early to report long-term outcomes. Patient-reported outcomes measures are unfortunately still not a part of the Danish Hernia Database, but it would be interesting to evaluate patient satisfaction including cosmetic results for robot-assisted and open repairs. There was a higher rate of pre-peritoneal mesh placement in patients undergoing open compared to robotic hernia repair. Information about the indication for the pre-peritoneal mesh placement was not available for the current study; however, this may be attributed to the fact that several hospitals in Denmark routinely use pre-peritoneal placement of flat mesh as the treatment of choice for primary ventral hernias. Although not common, some patients with larger ventral hernias may require skin resection as an adjunct to robotic ventral hernia repair, thus perhaps limiting the upside of the minimally invasive approach. The Danish Hernia Database holds no data about skin resection in minimally invasive procedures, and thus may represent a bias in the results. Hernia sac volume and potential loss of domain were not accounted for in the current study, as these variables were not available in the Danish Hernia Database. This potentially holds a bias, as patients with loss of domain more often undergo open repair in most centers in Denmark. Further, information about the site of the hernia (midline, sub-xiphoid, etc.) was not available in the current study.

## Conclusions

Robot-assisted ventral hernia repair is safe and feasible and is associated with shorter length of stay and decreased risk of readmission compared with open ventral hernia repair.

## Data Availability

Due to Danish law data is not available.
